# Surgical treatment of gastric stump carcinoma after Whipple procedure: A case report

**DOI:** 10.1097/MD.0000000000033808

**Published:** 2023-05-17

**Authors:** Wenjie Xie, Kuan Liu, Jianxiong Lai, Jian Li

**Affiliations:** a Department of General Surgery, The Third People’s Hospital of Mianyang Sichuan Mental Health Center, Mianyang, Sichuan, China.

**Keywords:** case report, gastric stump carcinoma, surgical treatment, Whipple procedure

## Abstract

Gastric stump carcinoma (GSC) is very exceptional and little known after Whipple procedure, and its diagnosis and treatment are extremely difficult to handle.

**Patient concerns:**

The patient, a 68-year-old man, visited our hospital’s General surgery outpatient clinic complaining of upper abdominal pain that had been bothering him for half a month. The endoscopy revealed lesions in the stomach residual, and the pathological results suggested adenocarcinoma. The patient underwent Whipple procedure for periampullary adenocarcinoma in the 4th year ago.

**Diagnoses:**

The final diagnosis was gastric adenocarcinoma and its pathological stage was Ⅱ A (T3N0M0).

**Interventions:**

The patient underwent stump gastrectomy and end-to-side esophagojejunostomy (Roux-en-Y reconstruction).

**Outcomes:**

The operation went smoothly and the patient recovered well with only mild bloating and nausea, and the symptoms completely disappeared during the hospital stay.

**Lessons:**

The development of GSC several years after Whipple procedure is uncommon. This is the first case from China that has received international attention. Early diagnosis is crucial. Surgery is considered to be the most effective treatment for GSC after Whipple procedure if long-term survival is possible and surgical risks are controllable.

## 1. Introduction

A century ago, Balfour proposed the influence of gastric reconstruction on the development of gastric stump cancer (GSC).^[1]^ In the later years, researchers had worked to clarify the pathophysiological mechanism behind the development of GSC. GSC was originally defined as gastric cancer (GC) that appeared from the remnant stomach more than 5 years after distal gastrectomy for benign diseases.^[2,3]^ But now, the current definition extends to the residual GC after gastric surgery for benign or malignant diseases in the stomach, which is also considered GSC.^[2,4,5]^ In general surgery, the Whipple procedure, a kind of pancreaticoduodenectomy, is regarded as one of the most challenging procedures due to its challenging operation and numerous postoperative complications.^[6]^ GSC is a common complication following surgery for GC, with an estimated incidence of 1% to 2%.^[7]^ However, it is extremely rare for GSC to occur after Whipple procedure for malignant tumors, and there are few reports of such cases in the English literature. Here we introduce a case that we believe to be an unusual case of 1 patient developing GSC 4 years after Whipple procedure for periampullary adenocarcinoma. This case is the first internationally reported 1 in China. We performed gastrectomy and gastrojejunostomy as a radical resection. The purpose of this article is to share our handling methods and accumulate information literature.

## 2. Case presentation

The patient, a 68-year-old man, visited our hospital’s General surgery outpatient clinic complaining of upper abdominal pain that had been bothering him for half a month. The patient had Whipple procedure for periampullary adenocarcinoma at the age of 64. This appointment is to treat stomach pain. His vital signs were steady upon arrival, with 121/76 mm Hg for blood pressure, 36.7 °C for temperature, 82/minutes for heart rate, and 20/minutes for respiration. An old surgical scar in the belly measuring 15 cm long and some minor pain in the left upper abdomen were discovered during a physical examination. Hemoglobin levels of 129 g/L (normal 130–175 g/L), serum albumin levels of 42.5 g/L (normal 40–50 g/L), hematocrit levels of 39.6% (normal 40–50%), C-reactive protein levels of 7.7mg/L (normal 0–5 mg/L), and a positive fecal occult blood test were among the abnormal laboratory findings. The outcomes of the remaining laboratory tests, including those for gastrointestinal tumor markers, were within normal ranges. It can be seen that the patient has an average nutritional level. The patient underwent Whipple procedure for periampullary adenocarcinoma 4 years ago. The postoperative histology and pathology revealed a moderately differentiated adenocarcinoma (International Union Against Cancer [UICC], T1bN0M0, stage Ⅰ A). The patient did not receive adjuvant chemotherapy after this surgery. For more than 20 years, he had been a smoker who smoked roughly 4 cigarettes every day and occasionally drank alcohol. There was no similar disease in the family.

**Figure 1.**
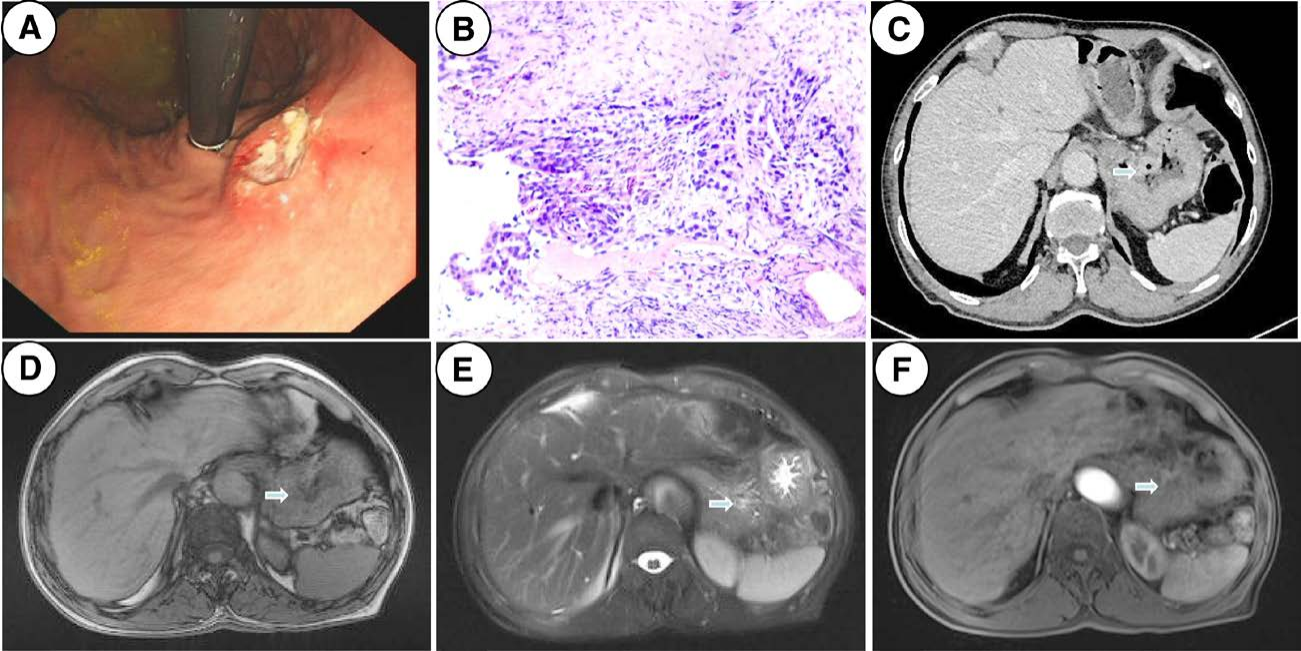
Preoperative examination data of the patient. (A) Gastroscopy showed that there was a 2cm^2^ ulcerative neoplasm near the cardia at the side of the lesser curvature of the stomach, and the edge of the neoplasm was volcanic, (B) pathological of endoscopic biopsy shows that the neoplasm was gastric adenocarcinoma (hematoxylin and eosin stain × 200 magnification), (C) CT scan of contrast medium injection: axial view (venous phase). It shows that the thickening of gastric wall at the cardia is slightly enhanced (arrows), and (D, E, F) magnetic resonance imaging showed that the gastric wall had varying intensity changes on T1WI, T2WI, and diffusion weighted imaging (DWI) (arrows). CT = computed tomography.

Gastroscopy revealed an ulcerative neoplasm near the cardia at the lesser curvature of the stomach, with a crater-like ridge at its edge (Fig. 1A). The pathological diagnosis of the neoplasm was adenocarcinoma. (Fig. 1B). A magnetic resonance imaging (MRI) scan of the upper abdomen and an abdominal computed tomography (CT) scan with an intravenous contrast agent both revealed slight thickening and enhancement of the stomach wall (Fig. 1C–F). It is worth noting that the patient’s stomach could not be filled with water during the imaging examination. The reason was that the pyloric barrier was broken because the remaining stomach was anastomosed with the jejunum during Whipple procedure. As a result, the structure of the stomach cannot be readily seen on CT or MRI images. However, we did not find any lymph nodes suspected of metastasis on the above imaging examination.

**Figure 2.**
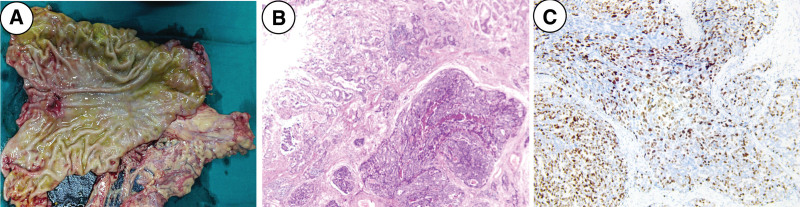
Postoperative examination data of the patient. (A) The resected remnant stomach specimen showed a tumor with a volume of 2 × 2 × 1cm^3^ near the cardia and biliopancreatic reflux (consistent with the findings of endoscopy), (B) pathological findings from resected specimens showed that moderately differentiated adenocarcinoma invades the lower serosa layer of stomach (hematoxylin and eosin stain × 400 magnification), and (C) Ki67 positive by immunohistochemical staining (hematoxylin and eosin stain × 400 magnification).

Based on the above aforementioned records, we considered that the patient’s tumor clinical stage was stage Ⅱ B (UICC, T3N0M0). Neoadjuvant therapy is not the first choice of treatment. All things considered, we carried out stump gastrectomy and end-to-side esophagojejunostomy (Roux-en-Y reconstruction) for the patient. Tumor tissue can be seen in the resected specimen (Fig. 2A). Histopathological examination revealed a poorly differentiated adenocarcinoma (UICC, T3N0M0, stage Ⅱ A) with the gastric subserous layer and peri-neural invasion but no vascular emboli. Immunohistochemical results: Ki-67 was approximately 40% (Fig. 2B and C), the expression of PCK and CD34 were positive, and CD56 and P53 were negative. No tumor metastasis was found in all 28 lymph nodes and greater omentum. The operation went well and the postoperative process was uneventful. The patient was released from the hospital after receiving follow-up care for 13 days, and no abnormalities were discovered during the subsequent routine follow-up inspection. The patient was very satisfied with the treatment, including the nursing care and surgical treatment. We recommend that the patient receive adjuvant chemotherapy after 3 months considering the patient’s physical condition. The patient has provided informed consent for publication of the case.

## 3. Discussion

According to the most recent GLOBOCAN data, GC is the fifth most common cancer worldwide. It continues to have a high incidence rate and mortality, which poses a major threat to human health.^[8]^ In an effort to aid in the prevention and treatment of GSC, additional research is being done to better understand the pathophysiological process underlying the occurrence and progression of GSC. In fact, experimental and clinical studies have always proved that chronic bile and pancreatic exocrine fluid return to the remnant stomach due to pyloric insufficiency, which may play an important role in the development of GC after Whipple procedure, or at least it is considered to be the cause of the histological changes of cancer precursors.^[9,10]^ The carcinogenic effect of biliopancreatic reflux on residual gastric mucosa is also indirect, including the increase of alkalinity of the residual stomach caused by atrophy of gastric gland mucosa, alkaline reflux is conducive to the growth of anaerobic nitrate-reducing bacteria and surgical inhibition of innervation of vagal intramucosal innervation. Schlag et al^[11]^ reported that bile pancreatic reflux can cause the colonization of carcinogenic bacteria, such as Escherichia coli. The nitrite produced by these bacteria will form carcinogens when contacting digestive proteins.^[12]^

Helicobacter pylori (*H pylori*) infection is known as the main pathogenic factor of gastric carcinogenesis. *H pylori* infection in the remnant stomach after gastrectomy is related to the pattern of gastritis, which is characterized by active and chronic inflammatory cells infiltrating the lamina propria. Research demonstrates that the existence of *H pylori* infection can modulate the severity of inflammatory alterations in the remaining stomach, which can result in chronic and acute inflammatory cell infiltration.^[13,14]^ In addition, it is speculated that eradication of *H pylori* in patients undergoing gastrectomy can prevent the occurrence of GSC. Furthermore, it was advised to eliminate *H pylori* infection in the gastric stump to prevent the occurrence of GSC in both the Second Asia Pacific Consensus Guidelines and the Maastricht IV/Florence Consensus Report.^[15,16]^ For this patient, endoscopic gastric mucosal examination showed that there was no *H pylori* infection, but bile and pancreatic juice reflux were confirmed to exist, which may be one of the causes of GSC. In addition, the absence of postoperative adjuvant chemotherapy and the expression of tumor genes may be the reasons that promote the occurrence of GSC. However, this is only a theoretical guess before the causal relationship is clear.

GSC is more challenging to diagnose than the entire stomach. One reason is that the clinical manifestations of GSC may be more insidious, with patients having basically no specific symptoms or only stomach discomfort. For example, some early and small lesions may not be visible through a double contrast examination of the upper gastrointestinal tract or by computed tomography. Second, doctors often do not think of GSC the first time when they receive such patients. In this case, after the first operation, the patient did not find any abnormality during long-term follow-up laboratory and imaging examinations. Similar to primary GC, endoscopy is still the preferred diagnosis method for GSC. It can take several samples of the tumor’s suspect regions and obtain pathology results. Endoscopic ultrasonography, CT, and MRI are still needed for tumor staging after the diagnosis of GSC, and the staging results will determine the different treatment modalities. A recent paper reported that more than 70% of GC was highly malignant undifferentiated cancer by studying the pathological characteristics of GC after pancreatoduodenectomy, which had a significant negative impact on the prognosis of patients. Since gastrectomy has been found to be a risk factor for GSC, the possibility of this complication should be kept in mind when evaluating patients with any type of malignancy who present with symptoms of recurrent metastatic disease after gastrectomy. Therefore, long-term follow-up is essential for patients after pancreaticoduodenectomy, which is conducive to early detection and timely treatment of GSC. Endoscopy should be considered as one of the most important tests during follow-up. According to a literature search, it had reported 36 cases (1 new case in our hospital) of GSC after pancreaticoduodenal surgery, and 35 of these cases were reviewed by Tatsuaki et al.^[17]^ We hope to make a small contribution to the case accumulation and treatment experience with this 1 case, which may be the first case ever published by China in the entire world.

Surgery may still be one of the main treatments for GC after Whipple procedure. For some early cases, surgery is the preferred method. Consistent with primary GC, surgery should include resection of the gastric stump and dissection of adjacent lymph nodes. However, the extent of lymph node dissection is controversial. Of course, neoadjuvant chemotherapy might be a smart choice in some advanced cases. After the local tumor is under control, additional surgical therapy can be more advantageous. In addition, it has been suggested that endoscopic submucosal dissection may be applicable to some early GC occurring in the remnant stomach.^[18]^ In this case, the patient did not receive postoperative adjuvant treatment after Whipple procedure and did not receive regular follow-up, which may also have a causal relationship with the occurrence of GSC. We performed stump gastrectomy and lymph node dissection according to the patient’s condition. Fortunately, postoperative pathology showed no lymph node metastasis.

## 4. Conclusion

It is rare for GSC to occur several years after Whipple procedure. Early diagnosis and treatment are critical. After gastrectomy, we should be alert to such complications, especially for patients with digestive tract symptoms. Endoscopy can be a crucial examination method for screening GSC. Once GSC is diagnosed, surgery is considered to be the most effective treatment for GSC after Whipple procedure if long-term survival is possible and surgical risks are controllable.

## Author contributions

**Investigation:** Jianxiong Lai.

**Supervision:** Kuan Liu.

**Writing – original draft:** Wenjie Xie.

**Writing – review & editing:** Wenjie Xie, Jian Li.
